# Editorial: Applications of non-invasive brain stimulation in neurodevelopmental disorders

**DOI:** 10.3389/fnhum.2026.1789798

**Published:** 2026-02-25

**Authors:** Aron T. Hill, Pushpal Desarkar

**Affiliations:** 1 Cognitive Neuroscience Unit, School of Psychology, Deakin University, Burwood, VIC, Australia; 2 Azrieli Adult Neurodevelopmental Centre, Centre for Addiction and Mental Health, Toronto, ON, Canada; 3 Department of Psychiatry, Temerty Faculty of Medicine, University of Toronto, Toronto, ON, Canada

**Keywords:** mental health, neurodevelopmental disorders, non-invasive brain stimulation, transcranial electrical stimulation, transcranial magnetic stimulation

Non-invasive brain stimulation (NIBS) technologies, including transcranial magnetic stimulation (TMS) and transcranial electrical stimulation, continue to evolve as powerful investigative and therapeutic tools across psychiatry, neurology, and cognitive and clinical neuroscience. NIBS enables safe *in vivo* perturbation of brain circuits, providing information on cortical excitability and inhibition. When combined with functional brain recordings, it also assists assessment of network propagation, connectivity, and brain oscillatory dynamics (Deng et al., 2020; Ziemann et al., 2026). Moreover, plasticity-inducing NIBS protocols, which produce enduring modification of neural activity within targeted brain regions and networks, can also be used to help draw a level of causal inference about the role of specific brain regions and/or networks to cognition and behavior (Polanía et al., 2018).

NIBS technologies also hold significant utility as a means of therapeutically modifying disrupted brain circuits across a growing range of clinical conditions. However, despite continued methodological advances, the application of NIBS in neurodevelopmental disorders remains relatively limited. Neurodevelopmental disorders comprise a broad and heterogeneous group of conditions including autism spectrum disorder, attention deficit/hyperactivity disorder, communication and motor disorders, as well as intellectual disability and specific learning disorder (American Psychiatric Association, 2022). These conditions impact a substantial proportion of the global population and are frequently associated with enduring impacts on an individual's health, everyday functioning, and quality of life (World Health Organization and UNICEF, 2023). However, progress toward effective, neurobiologically-focussed therapies has been constrained by the complex and heterogeneous etiology of these conditions, as well as by the fact that their underlying neurobiological mechanisms remain incompletely understood (Astle et al., 2022; Thapar et al., 2017). In this editorial, we introduce a collection of conceptual and empirical articles that explore some key emerging applications of NIBS in neurodevelopmental disorders.

In a perspective article, Desarkar discuss the increasing evidence for hyper-plasticity as a potential key underlying feature in autism that could adversely affect cognitive and behavioral outcomes. A testable framework for assessing and modifying neuroplasticity in autism using TMS is outlined, including a novel approach to assess altered neuroplasticity across both motor and non-motor brain regions. An innovative intervention strategy is then proposed, involving administration of bilateral “mechanism driven” high-frequency repetitive TMS (rTMS) targeting either the primary motor cortex (M1), sensory cortex (S1), or dorsolateral prefrontal cortex (DLPFC). If successful, such an approach, designed to diminish hyper-plasticity, could serve to improve motor, sensory, and executive function difficulties in autistic adults.

Shifting focus to dyslexia, Gallagher et al. present a hypothesis article proposing an approach to model dyslexia in neurotypical adults using a combination of neuroimaging (structural and functional MRI) and neuromodulation via transcranial temporal interference stimulation (tTIS). The authors suggest that this approach could be used to first cluster individuals into neuropathology-derived dyslexia subgroups based on neuroimaging findings, and then, using tTIS, down-regulate activity in regions identified by the clustering analysis with the aim of inducing transient subtype-specific symptoms in neurotypical individuals. If successful, this approach could inform the development of more personalized therapeutic neuromodulation strategies for dyslexia.

Two independent studies in this Research Topic address important safety and feasibility aspects of NIBS. In an open-label pilot trial, Fraser et al. evaluate the safety, feasibility, and tolerability of bi-hemispheric transcranial direct current stimulation (tDCS) applied over the left and right motor cortices and paired with rehabilitation therapy in childhood-onset stroke survivors. tDCS was found to be well tolerated, with no major adverse events reported. Common sensations associated with stimulation included self-limited itchiness or tingling (40% of sessions). Some improvements in average upper extremity function and performance were also noted but require cautious interpretation as inferential statistics were not performed due to the small sample size and feasibility design of the study.

In a separate study, Collins et al. present a longitudinal evaluation of the safety, tolerability, and feasibility of single-pulse TMS in infants with perinatal brain injury. Infants completed between one and four neuro-navigated TMS sessions with stimulation applied over M1 and surface electromyography (EMG) recorded from wrist flexor muscles. No adverse events were reported, and no significant changes in heart rate or respiratory rate were observed. Longitudinal retention rates were also high (85%), and motor-evoked potentials (MEPs) were successfully recorded in 95% of participants. Collectively, these findings help support the feasibility of TMS in neurodevelopmental research, indicating that protocols can be well-tolerated and yield meaningful neurophysiological outcomes.

Finally, Conelea et al. used single-session rTMS to probe the functional contribution of the supplementary motor area (SMA) in youth (12–17 years) with Tourette syndrome. Using MRI-guided neuro-navigation, participants received acute inhibitory low-frequency (1 Hz) rTMS or sham stimulation in a randomized, sham-controlled design, with tic expression, voluntary tic control, and premonitory urge intensity assessed both before and after stimulation. Active rTMS was associated with modest reductions in natural tic frequency, and lower premonitory urge intensity during suppression, alongside improvements in tic controllability. Due to a small sample size (*N* = 14), analyses used descriptive statistics and effect size estimation; however, they nevertheless provide important preliminary feasibility and mechanistic support for rTMS targeting the SMA in Tourette syndrome.

Collectively, these articles underscore the valuable role of NIBS technologies in interrogating and modulating brain circuits in neurodevelopmental disorders. The contributions by Fraser et al., and Collins et al., help to strengthen the field's evidence-base around safety and feasibility of these techniques in child and adolescent cohorts. Desarkar and Gallagher et al. outline innovative frameworks with potential to advance therapeutic neuromodulation in autism and dyslexia, respectively; while Conelea et al., provide preliminary evidence that the SMA can be targeted with rTMS in youth with Tourette syndrome to influence behavioral outcomes.

These contributions provide a meaningful advance in NIBS research and highlight the emerging innovation surrounding neurostimulation methodologies in neurodevelopmental disorders. Looking ahead, it will be important for research to build on these foundations to further develop robust NIBS protocols for biomarker discovery and therapeutic interventions for individuals with neurodevelopmental disorders. Key priorities include the continued refinement of precise (and individualized) structural and functional targets, as well as the development of well-powered sham-controlled clinical trials. Further integration of neurophysiological techniques such as electroencephalography (EEG), TMS-EEG, and magnetoencephalography (MEG) will also be valuable for comprehensively quantifying stimulation target engagement and identifying effective treatment sites.

## Data Availability

The original contributions presented in the study are included in the article/supplementary material, further inquiries can be directed to the corresponding author.
